# Urban-Rural Dimension of Falls and Associated Risk Factors among Community-Dwelling Older Adults in West Java, Indonesia

**DOI:** 10.1155/2021/8638170

**Published:** 2021-08-20

**Authors:** Susiana Nugraha, Sabarinah Prasetyo, Indri Hapsari Susilowati, Tri Budi W. Rahardjo

**Affiliations:** ^1^Doctoral Candidate, Faculty of Public Health Universitas Indonesia, Jakarta, Indonesia; ^2^Faculty of Health Sciences, Universitas Respati Indonesia, Jakarta, Indonesia; ^3^Department of Biostatistics, Faculty of Public Health Universitas Indonesia, Jakarta, Indonesia; ^4^Department of Occupational Health, Faculty of Public Health Universitas Indonesia, Jakarta, Indonesia

## Abstract

Falls are one of the common problems among older adults; it is estimated that 684,000 fatal cases of falls occur every year. Furthermore, falls constitute one of the leading causes of mortality due to accidental injury. This study aims to identify the risk factors for falls in the older adults who live in the community, according to the dimensions of the living area: in urban and rural. The proportional sampling method was used to identify the rural and urban areas in West Java Prefecture; meanwhile, the incidence of fall in the last 12 months was selected as the outcome variable. Furthermore, sociodemographic background, chronic medical condition, fear of falling, visual and hearing impairments, Activity of Daily Living (ADL), Barthel index, physical performance (Short Performance Physical Battery (SPPB)), and living environment were analyzed to identify the risk factors that contribute to the incidence of falls. A total of 611 older adults participated in this study: 62% of them are living in rural area and 38% of them are living urban areas. More than 70% of study participants were aged 60–69 years, while 73% were females. There is no significant difference in fall prevalence in rural (16.5%) and urban (10.7%) areas (*p* value = 0.228). Furthermore, the multiple logistic regression analysis showed that the male gender (OR = 0.29, 95%CI [0.09–0.88]), chronic illness (OR = 3.25, 95%CI [1.24–8.53]), and visual impairment (OR = 3.6, 95%CI [1.52–8.54]) were associated with fall among older adults in urban areas. Meanwhile, visual impairment (OR = 1.81, 95%CI [1.03–3.18]) and living environment (OR = 3.36, 95% CI [1.14–9.93]) were significantly associated with falls in rural areas. Based on the associated risk factors identified in this study, a different approach is needed to reduce the falling risk among older adults in urban and rural areas in Indonesia.

## 1. Introduction

The aging process significantly influences older adults' physical and functional capacity and is associated with weaknesses, limitations, and disabilities. This condition affects balance and increases falling risk; meanwhile, fall-related injury has been identified as a major public health issue. Furthermore, older adults have a higher falling risk due to the decreased functional body motor and cognition [1–4].

Falls are one of the common problems among older adults; it is estimated that 684,000 fatal falls occur each year, making it the second leading cause of unintentional injury death, after road traffic injuries [5]. Therefore, falls constitute one of the leading causes of mortality in older adults due to accidental injury. Furthermore, over 80% cases of falls in low- and middle-income countries amount to more than two-thirds of the highest deaths among adults aged 60 years and above [6]. The Indonesian National Health survey (2018) reported that the proportion of injuries caused by falls among the age group of 55 years and above is 25%; meanwhile, 67% occur within the home environment [7].

In 2019, the percentage of the older adult population reached 9.60%, or approximately 25.64 million [8]. This shows that Indonesia is transitioning towards an aging population because the percentage of adults aged 60 years and above is more than 7% of the total population. At 10%, the country is expected to have an aging population structure. Given such a large population, there is a need to explore the risk factors associated with accidental falls and deterioration of health. Meanwhile, the various consequences of falls in older adults include post-fall anxiety syndrome, injury to either soft tissue or broken bones, hospital treatment, disability (decreased mobility), decreased functional status or dependency, increased use of health care facilities, and ultimately death [9–12]. In addition, the adverse effect of falls might significantly influence mobility, thereby decreasing the quality of life up to 70% [13].

Fall-related risk among community-dwelling older adults differs from assessment in a hospital or long-term care facility setting. Older adults in the community are usually more active and physically independent compared to others in long-term care facilities. Nevertheless, the assessment needs to be multifactorial, due to the complex nature of falls. Meanwhile, multiple factors have been shown to increase fall risk in community-dwelling adults aged 65 years and above. These factors are divided into 3 categories, namely, demographic (age and gender), intrinsic including health conditions such as the existence of chronic illness [14, 15], history of falling [16–18], functional capacity which refers to the level of dependency in performing daily activities, and physical capacity indicated by gait speed and balance examination [19–23], sensory impairment such as visual and hearing impairment [24–27], and depression [28–30]. Finally, the extrinsic factors include living environment, home hazard, neighborhood accessibility [22, 31], and types of medication consumed [32, 33].

Given the longstanding disparities in rural and urban areas, this study aims to investigate the rural-urban dimension of falls among the Indonesian older adult population. The differences between both environments determine the types of fall risk faced by adults. These include population density, percentage of agricultural households, and the presence or access to urban facilities such as roads, formal education, and public health facilities, etc. [34]. The Indonesian older adult population is widely distributed with a higher population (52.8%) in the urban compared to rural areas (47.20%) [8]. Furthermore, the living environments for rural and urban older adults are also dissimilar. For example, most rural residents live in wider one-story buildings, while urban residents live in densely populated buildings with more than one-story buildings. Moreover, access to public services is easier for urban residents, such as good transportation facilities, while rural residents exercise more efforts such as walking through areas with hilly topography. The dissimilar fall incidence in both areas is influenced by diverse environmental factors, cultural traditions, and leisure and physical activities. Based on the evidence presented in the previous literature, this study hypothesizes that rural-urban disparities lead to varying patterns and determinants of falls among older adults.

Although numerous studies have identified factors associated with falls, only a few examined the rural-urban dimensions among Indonesian older adults. In addition, no study has compared the incidence and risk factors of falls among urban and rural older adults in the country. In more detail, this study aims to identify prevalence of falls in and the risk factors for falls in the older adults who live in the community dwelling in urban and rural areas. A clearer understanding of the risk factor for falls in older adults is essential for elucidating prevention strategies.

## 2. Materials and Methods

### 2.1. Study Design

This study uses a cross-sectional analytical approach to identify the incidence of falls and to investigate the risk factors of falls associated with the fall incidence at the same time.

The household survey was carried out from June to September 2020. Data collection activities include structured interviews, physical performance test, and home environment observation. All data collection was conducted by a research enumerator who had previously been trained by researchers.

### 2.2. Population and Samples

The study population were community-dwelling older adults aged 60 years and above. The sample size was calculated based on the prevalence of 29% in the previous year [35], and the precision was considered as 95% (±0.05) for each area. Meanwhile, the West Java Provinces were selected, being one of the Indonesian provinces transitioning into an aging population, i.e., having more than 10% older adults [8]. Furthermore, a cluster sampling method was used to obtain the required study sample from 2 selected areas out of 28 municipalities in the West Java administrative area, namely, Bandung Regency representing the rural area and Bandung City representing the urban area. Four subdistricts were then randomly selected from each rural and urban area, while 2 villages each were randomly selected from the subdistrict. Therefore, a total of 16 rural and urban villages were selected. The sample size for each village was calculated proportionally, and the sampling was carried out randomly. The sample selection based on the inclusion criteria in this study included age 60 years or older, able to communicate well, did not have severe dementia, did not experience severe mental disorders, was not in bed-ridden condition, and was willing to be a respondent in this study.

### 2.3. Ethical Consideration

This study followed the Helsinki Convention's norms and later modifications as well as the uniform requirements for manuscripts submitted to biomedical journals. The researcher ensured that the data collection process fully respected and protected personal privacy. Informed consent was provided by participants before engaging with the questionnaire and the survey was conducted anonymously to protect respondents' privacy. Before participation, the respondents voluntarily signed written informed consent; meanwhile, ethical clearance was obtained from the Institutional Research Bureau, Faculty of Public Health University of Indonesia, with approval number: Ket- 326/UN2.F10.D11/PPM.00.02/2020.

### 2.4. Variables and Measures

#### 2.4.1. Fall

Fall is the major outcome of this study. The definition of fall is in accordance with WHO definition of fall as an event which results in a person coming to rest inadvertently on the ground or floor or other lower level. [6] Fall in this study was assessed with the following questions: “have you fell in the last twelve months?” and “how many times have you fallen?”

#### 2.4.2. Risk Factors for Falls

The sociodemographic factors include age, sex, education, occupation status, and residential area. Meanwhile, chronic medical conditions were assessed by asking the question “have you been diagnosed for the following diseases by a doctor/paramedic/nurse/midwife?” (“hypertension, diabetes or high blood sugar, heart diseases, stroke, arthritis/rheumatism, and depression”). The subject is considered as having chronic illness when any of the medical conditions is found.

Visual impairment refers to any degree of defect in a person's vision [36]. It was assessed subjectively with the question “do you have any visual problems such as blurred vision? (yes/no). The answers were then evaluated with the tumbling E chart test to confirm subjective visual impairment. Moreover, corrections with glasses were considered as normal vision.

Hearing impairment refers to a defect in the ability to hear, as well as normal hearing at thresholds of 20 dB and above in both ears [37]. This was assessed using the question “do you have any problems with your hearing function that interferes with your daily activities?” (yes/no). The answers were then confirmed by observation and whisper test. Moreover, corrections with hearing aids were considered as normal hearing.

Functional capacity was measured by Barthel index of ADL (Activity of Daily Living). This index measures the likelihood of living at home with a degree of independence [38]. Ten basic activities of daily living (ADL) were captured, namely, bowels, bladder, grooming, toilet use, feeding, transfer, walking, dressing, climbing stairs, and bathing. A dichotomous category was selected as mild/severe dependent ≤19, and independent score 20 [38, 39].

The physical performance measured by SPPB (Short Physical Performance Battery) is a well-known and sensitive test which combines the results of 3 balance tests, a gait speed, and chair stand test into one score [40, 41]. Hand-clocked durations are used to indicate the performance capacity for individual components, which were then scaled to a grade between 0 and 4 and added up to a score between 0 and 12. The result was then dichotomized using ≤9 into poor and >9 into good physical performance [41]. All of the tests were performed by a trained assessor.

Living environment was assessed with the CDC Home Fall Prevention Checklist [42] for older adults to identify home hazard that increases the risk of falling. Home environmental observation checklist had been conducted by trained assessors. The checklist included room lighting, floor condition, stairs and steps, kitchen, bedroom, and safety in the bathroom. A “yes” for any of the hazards indicates an unsafe environment.

### 2.5. Statistical Analysis

The data were analyzed using statistical IBM SPSS Statistics for Windows, version 25.0 (IBM Corp., Chicago, IL, USA). A descriptive analysis was performed to describe the sociodemographic profile of the participants. Furthermore, the independent sample *t*-test and chi-square analysis were used to compare the falling risk among the two groups (rural and urban). Subsequently, binary multiple logistic regression analysis was performed to identify the association between falls and risk factors.

## 3. Results

This study was conducted in 2 representative rural and urban areas in West Java, Indonesia. A total of 611 samples consisting of 230 and 381 from urban and rural areas, respectively, participated in this study with a 77% response rate.

Table 1 shows that 19% of the respondents had a history of falls in the last 12 months, and there is no significant difference between residents in the rural and urban areas. Participants living in the rural area were significantly older compared to the urban residents, while hearing and visual impairment were significantly higher in the rural areas. Furthermore, rheumatoid/osteoarthritis was found in 59% of the participants, while the majority were also found to be living in an unsafe environment. Older adults in rural areas had significantly higher cases of unsafe living compared to urban. In addition, significantly poorer functional capacity and physical performance were found in the rural areas; meanwhile, there was no significant difference in the frequency of falls between both areas.

Table 2 shows the logistic regression analysis results for the variables associated with falls among older adults living in rural and urban areas. There was a significant association between gender and falls among urban older adults after adjusting with other risk factors; males were likely not to fall compared to females (AOR = 0.29, 95%CI [0.09–0.88]). Furthermore, visual impairment was significantly associated with the incidence of falls in both areas, while older adults in the urban area with visual impairment had 3 times higher risk of falls (AOR = 3.60, 95% CI [1.52–8.54]). In contrast, older adults in rural areas with visual impairment were likely to fall 1.8 times compared to residents with normal vision (AOR = 1.81, 95%CI [1.03–3.18]). The chronic medical condition was only associated with the incidence of falls in urban areas, with 3 times higher falling compared to healthy older adults (AOR = 3.25; 95%CI [1.24–8.53]). Furthermore, the living environment was associated with the incidence of falls in the rural area. Older adults in unsafe environments were likely to fall 3 times more than others in safe environments (AOR = 3.36 95%CI [1.14–9.92]).

## 4. Discussion

This study sought to identify the different risk factors associated with falls among community-dwelling older adults in urban and rural areas using the multifactorial approach. The findings suggest that more falls occurred in the urban area (16.5%) compared with the rural area (10.7%) (although it did not show any statistically significant association). The incidence of falls in the urban area is influenced by gender (male), visual impairment, and chronic illness. Meanwhile, visual impairment and safety of the environment were significantly associated with falls in rural areas.

Both urban and rural elderly share visual impairment as risk factor of falling. Visual impairment is characterized by subjective complaints of vision acuity that affects the daily living activities. Vision problems may increase the risk for falling because of obstacle avoidance based on diminished perception of spatial relationships and distances. The older adults who have visual impairment will have difficulty identifying the risk of danger that may result in falling in front of them. This study identified older people with visual impairment who are living in urban areas likely to fall 3.6 times higher than those with normal vision. Compared to those living in rural area, the urban older people have higher odds ratio (AOR = 3.60, 95% CI [1.52–8.54]) to falls compared to rural group (AOR = 1.81, 95%CI [1.03–3.18]). This finding can be assumed that the urban environment is generally a densely populated building, and the houses have more than one floor and norrower housing environment causing the elderly to have greater risk of falling. This condition leads the elderly who have visual impairments to have difficulty identifying the potential hazards that cause falls. Several studies have identified the association between visual impairment and falls. The Shinpay Eye Study community in China showed that fall incidence was significantly associated with Snellen's visual acuity, which was best corrected to less than 6/12. Furthermore, a study in Shenzhen, China, showed that impaired vision increased falling risk [27]. A longitudinal study by Hong et al. stated that older adults with visual impairment are likely to experience more falls [43].

Gender has been identified as one of the risk factors associated with falls among older adults in urban areas. Based on the results, male older adults are likely to have lower risk of falls compared to females. This gender disparity of falls may be due to differences in higher levels of physical activity, muscle strength, and bone density in men than in women. This finding is in line with a study conducted by Zhang et al. who stated that males in urban areas have a lower risk of falls compared to females among Chinese older adults [44]. Male elder people in urban areas are mostly retired from their formal jobs. They are used to actively working, and likely have good residual muscle strength to support the body steadily; hence it is beneficial as a protective factor for falls [45]. On the other hand, the older women in urban area are mostly housewives who have had monotonous activities for years and likely have poorer muscle strength and prone to frailty; hence they are at risk of falling.

Chronic illness is widely common among older adults in both urban and rural areas. Chronic illness refers to degenerative diseases experienced as part of the aging processes. Although the prevalence of chronic illness in rural areas is significantly higher compared to urban areas, the direct association between chronic illness and fall was only found in the urban model in single association, even showing higher odd ratio after adjusting with other risk factors. Older adults in urban areas with chronic illness have a 3 times higher risk to fall than who did not have chronic illness. Chronic illness has significant impact in deteriorating physical function that increases the risk of falls [46–48]. This condition is exacerbated by poor physical activity following retirement among the urban elderly. Moreover, there is easier access to transportation and public facilities in urban areas, causing activities that involve physical exercise such as walking that is beneficial for strengthening the lower limbs in older people to be rarely carried out. Urban communities tend to have a higher level of stress from both the society and the environment hence affecting the quality of body rest. If this condition is experienced by the elderly who have chronic diseases, it will decrease the body's functional capacity so that it can increase the risk of falling. Meanwhile, chronic illness is widely known to significantly affect the physical and functional capacity of older adults. It affects balance and posture stability, thereby increasing the falling risk [49].

The results also showed that unsafe living environments increase falling risk 3 times higher among older adults in rural areas. An environment is considered unsafe when it negatively affects the performance of daily activities. Approximately 96% of older adults in rural areas live in an unsafe environment, thereby increasing the falling risk. This result is in line with Zhang et al.'s study which reported a significant association between living environment and the incidence of falls among older adults in the rural areas [44]. The use of shared communal latrines placed outside the building is more common in the rural areas. People in rural areas generally have a large area of land, so they tend to build large houses with separate rooms. They build a house with two or three ridges including a separate kitchen. In addition to building houses, other equipment is needed that forms a single ridge even though they are of different sizes, such as rice granaries, firewood storage areas, rice pulverizers, cattle pens, and so on. Rural communities generally use shared water sources with other residents for bathing and latrine purposes. Therefore, older adults in the rural areas need to leave the house to access toilets and clean water. The above conditions are quite difficult for the older adults to carry out daily activities, especially in old conditions. The topography of the rural area which is contoured and the condition of the houses that have many obstacles increase the risk of balance disturbances that result in falls.

The limitations include the ability to consider covariates affecting the study outcomes. First, the data were based on a cross-sectional survey; therefore, the study was unable to separate the cause-effect relationships of variables. In addition, recall bias might exist, especially among older adults with poor memory. Given that the data were obtained in 12 months, the frequency of falls might be underreported due to the longer study period. Furthermore, the measures of environmental factors are also scarce; they are based on observation during data collection; hence, behavior towards the living environment was not assessed. Therefore, further studies are needed to comprehensively explore fall risk factors.

Notwithstanding, this is one of the first studies that analyzed rural-urban dimension of falls and its associated risk factors among Indonesian community-dwelling older adults. Therefore, the results constitute an important basis for future prospective studies and possible fall prevention programs for older adults. This is nevertheless an important finding given the sparse data available for rural and urban comparison and will go on to inform future prospective studies to confirm these findings. In addition, this study also provides useful implications for other countries with similar characteristics, such as the countries in Southeast Asia.

## 5. Conclusion

This study identified the different risk factors associated with falls among older adults based on the characteristics of the study area. These results suggest the need for a different approach to reduce the incidence of falls among older adults. Therefore, the government and local agencies need to develop suitable prevention strategies using rural and urban approaches, especially concerning gender, visual impairment, existing chronic illness, and living environment.

## Figures and Tables

**Table 1:** Characteristics of study participants.

Variables	Urban (*n* = 230) *n* (percent)	Rural (*n* = 381) *n* (percent)	Total	*p* value
*Ages*				
60–69 years	156 (67.8%)	271 (71.1%)	427 (69.9)	0.001
70–79 years	68 (29.6%)	72 (18.9%)	140 (22.9%)	
80 years and above	6 (2.6%)	38 (10%)	44 (7.2%)	

*Gender*				
Male	51 (22.2%)	113 (29.7%)	164 (26.80%)	0.756
Female	179 (77.8%)	268 (70.3%)	447 (73.20%)	

*Current occupation*				
Actively working	32 (13.9)	172 (45.1%)	204 (33.4%)	0.001
Not working	198 (86.1%)	209 (54.9)	407 (66.6)	

*Visual impairment*				
No	175 (76.1%)	157 (41.2%)	332 (54.3%)	0.001
Yes	55 (23.9%)	224 (58.8%)	279 (45.7%)	

*Hearing impairment*				
No	185 (30.3%)	302 (49, 4%)	487 (79.7%)	0.001
Yes	25 (4.1%)	99 (16.2%)	124 (20.3%)	

*Chronic medical condition*				
Hypertension	89(38.7%)	174 (45.7%)	263 (43%)	0.124
Stroke	8 (3.5%)	9 (2.4%)	17 (2.7%)	0.236
Heart diseases	16 (7.0%)	12 (3.1%)	28(4.5%)	0.028
Diabetes	30 (13%)	23 (6%)	53 (8.6%)	0.003
Rheumatoid/osteoarthritis	112 (48.7%)	250 (65.6%)	362 (59%)	0.001
Parkinson	3 (1.3%)	27 (7.1%)	30 (4.9%)	0.003

*Having unsafe home environment*				
No	24 (10.4%)	12 (3.1%)	36 (5.90%)	0.001
Yes	206 (89.6%)	369 (96.9%)	575 (94.10%)	

*The need for ADL support*				
Independent	199 (86.5%)	317 (83.2%)	516 (85.30%)	0.05
Dependent	25 (10.9%)	64 (16.8%)	89 (14.70%)	

*Physical performance*				
Good	123 (53.5%)	122 (32%)	245 (40.10%)	0.001
Poor	107 (46.5%)	259 (68%)	366 (59.90%)	

*Fall in the last 12 months*				
No	192 (83.5%)	303 (79.5%)	495 (81%)	0.228
Yes	38 (16.5%)	78 (10.7%)	116 (19%)	

*Frequency of fall*				
1 fall	27 (71.1%)	51 (65.4%))	78 (67.2%)	0.528
2 or more falls	11(28.9%)	27 (34.6%	38 (32.8%)	

**Table 2:** Risk of falling among older adults in urban and rural areas.

Risk factor for fall	Urban		Rural	
Variables	Crude OR; 95% C. I.	Adjusted OR; 95% CI	Crude OR; 95% CI	Adjusted OR; 95% CI
*Gender*				
Male	1.525 (0.749–3.106)	0.289 (0.095–0.882)^*∗*^	1.349 (0.762–2.387)	1.004 (0.545–1.849)
Female (ref)	1		1	1

*Ages*				
60–69 years	0.821 (0.092–7.363)	1.213 (0.116–12.645)	0.858 (0.385–1.916)	0.917 (0.385–2.184)
70–79 years	1.415 (0.153–12.058)	2.385 (0.223–25.450)	0.644 (244–1,702)	0.659 (0.241–1.806)
80 years old and above (ref)	1	1	1	1

*Current occupation*				
Actively working	0.835 (0.318–2.193)	0.416 (0.131–1.322)	0.950 (0.577–1.566)	0.873 (0.497–1.533)
Not working (ref)	1	1	1	1

*Visual impairment*				
No (ref)				
Yes	2.478(1.185–0.183)^*∗*^	3.601(1.517–8.544)^*∗∗*^	1.896(1.108–3.244)^*∗*^	1.807(1.027–0.179)^*∗*^

*Hearing impairment*				
No (ref)				
Yes	1.959 (0.802–4.787)	1.538 (0.554–4.271)	1.392(0.799–2.425)	1.214 (0.674–2.187)

*Chronic medical condition*				
No chronic illness (ref)	1		1	
With chronic illness	2.657 (1.112–6.347)^*∗*^	3.247 (1.236–8.533)^*∗∗*^	1.652 (0.845–3.231)	1.516 (0.761–3.019)

*Physical performance*				
Good	1		1	
Poor	2.329 (0.524–10.352)	0.545 (0.166–1.788)	0.765(0.202–2.897)	0.98 (0.485–1.98)

*The need of ADL support*				
Independent (ref)	1	1	1	1
Dependent	.766(0.268–2.191)	1.022 (0.468–2.231)	1.012 (0.519–1.972)	0.792 (0.458–1.369)

*Living environment*				
Safe	1	1	1	1
Unsafe	704 (0.335–1.480)	.51 (0.219–1.192)	3.636 (1.276–0.357^*∗∗*^)	3.358 (1.137–9.921)^*∗∗*^

*Note.*^*∗∗*^*p* ≤ 0.01^*∗*^*p* ≤ 0.05; OR = odd ratio; CI = confidence interval.

## Data Availability

The datasets generated and analyzed in this study are not publicly available because ethical guidelines prohibit researchers from providing research data to third-party individuals.
